# Quantum-Confined Stark Effect in Polar InGaN/GaN Quantum Wells of Different Widths Studied by Photoluminescence Under Hydrostatic Pressure

**DOI:** 10.3390/ma19122473

**Published:** 2026-06-09

**Authors:** Tadek Suski, Grzegorz Staszczak, Witold Trzeciakowski, Lukas Uhlig, Jannina Jacqueline Tepaß, Mateusz Hajdel, Grzegorz Muzioł

**Affiliations:** 1 Institute of High Pressure Physics, Polish Academy of Sciences, 01-142 Warsaw, Poland; tadek@unipress.waw.pl (T.S.); staszczak@unipress.waw.pl (G.S.); hajdel@unipress.waw.pl (M.H.); gmuziol@unipress.waw.pl (G.M.); 2 Institute of Physics, Chemnitz University of Technology, 09126 Chemnitz, Germany; lukas.uhlig@physik.tu-chemnitz.de (L.U.); jannina-jacqueline.tepass@physik.tu-chemnitz.de (J.J.T.)

**Keywords:** InGaN quantum wells, high pressure, photoluminescence, modeling of PL spectra

## Abstract

Low-temperature photoluminescence (PL) has been studied under hydrostatic pressure and varying excitation powers in three samples of single In_0.17_Ga_0.83_N quantum wells with different widths: 2.6 nm, 5.2 nm, and 10.4 nm. Transitions involving ground states were strong in the 2.6 nm well, weak in the 5.2 nm well, and absent in the 10.4 nm well. Pressure coefficients of PL lines have been used to estimate the electric field in the wells. In the widest well, the field seems to be fully screened (at high excitation powers). Simulations involving Poisson and Schrödinger equations allowed us to identify the experimental PL lines. Pressure evolution of the PL spectra agreed with the simulation. We present diagrams showing the dependence of the field in the well on pressure and on carrier concentration. In wide wells, these diagrams illustrate the transition from a 2D-like system to a 3D-like system.

## 1. Introduction

InGaN/GaN quantum wells are the building blocks of optoelectronic devices operating in the blue-green spectral region. Recent successes in the design of efficient nitride emitters make it possible to achieve external quantum efficiencies approaching unity [1,2,3]. There are also attempts to obtain efficient devices operating in red [4,5], green [6] and UV [7,8] regions of the spectrum. The corresponding multilayer structures and devices are generally grown epitaxially along the hexagonal polar crystallographic c-direction. They are characterized by strong spontaneous and piezoelectric polarizations, the latter induced by the omnipresent lattice mismatch between the substrate and individual layers of these heterostructures. The presence of epitaxial strain leads to an increase in the built-in electric field *F_int_* [9,10,11]. Strong field causes the so-called Quantum-Confined Stark Effect (QCSE) [12,13], consisting of the lowering of wavefunction overlap (between electrons and holes) and the red shift of optical transition energies. The magnitude of this shift is dependent on the internal electric field, *F_int_*, and on the width of the quantum well, *d*.

The QCSE causes great changes in the band structure of polar quantum wells (QWs grown along the <0001> direction) and consequently in optoelectronic devices based on them. The profiles of the conduction and valence band edges in the QWs show tilting. Electrons and holes are pushed towards the opposite edges of the QW. The reduced overlap of the electron and hole wave functions results in a drastic reduction in luminescence intensity. The additional disadvantage of the presence of QCSE in the active region of nitride emitters is the sensitivity of their emission wavelength/energy to the magnitude of the applied electrical or optical excitation, i.e., to the change in the applied driving current density or laser power density. This effect is due to the screening of the built-in field and results in poor control of the emitted light wavelength. Furthermore, in the case of electroluminescence, increasing the driving voltage introduces an increase in the built-in field in the light-emitting diodes (LEDs) or laser diodes (LDs).

It is worth mentioning that, despite the negative effects arising from the presence of the mentioned above *F_int_* in polar InGaN/GaN structures and devices, there are also examples of using QCSE to improve their performance. A well-known example is the AlGaN/GaN field effect transistor with a 2-dimensional electron gas of high mobility [13,14]. Another positive example is the polarization doping in UV LEDs [15,16].

To minimize the detrimental effects of the internal polarization, growing devices along orientations with zero or minimal polarization has been proposed (see, e.g., [17]). However, it should be noted that until recently, all attempts to construct highly efficient optoelectronic devices on such non-polar or semipolar directions of epitaxial growth have failed. The reason is the poor crystallographic quality of such structures and devices.

In commercial LEDs and LDs, the common range of QW width is 2–3 nm. Such narrow QWs enable the maintenance of a high electron and hole wave function overlap and thus reasonable electroluminescence intensity [18,19].

For several years now, there has been information about attempts to reduce the polarization caused effects by using wider InGaN/GaN QWs [20,21]. Apart from academic studies, this solution has also been tested by the industry [20,22]. It was shown that radiative recombination in LEDs with 5 nm and 20 nm wide QWs is strong and, for the most part, originates from excited quantum well states.

In the present work, we focus on the studies of the photoluminescence generated in the QW structures representing the following three cases: narrow (2.6 nm), intermediate (5.2 nm), and thick (10.4 nm) In_0.17_Ga_0.83_N/GaN QWs. We observe three corresponding regimes of photoluminescence generation. The current understanding of the operation of such quantum structures can be compared with the results obtained from earlier studies of emitters (LEDs and LDs) [21].

In the present paper, we study low-temperature PL on samples without a *pin* junction. PL is superior to EL since EL can only be measured at high temperatures. At low temperatures, we see more transitions. We also present more advanced interpretation (developed in [23]), showing multiple transitions in wider wells, and present diagrams showing the electric field as a function of pressure and carrier concentration. In wide wells, these diagrams reveal a transition from a 2D-like to 3D-like system, while for narrow wells, the system is always 2D-like. The dependence of PL spectra on laser power density leads to the blue shift of the observed transitions due to the screening of the electric field by free carriers. On the contrary, high pressure increases the built-in electric field [24,25]. It also increases the bandgap of GaN and InGaN. The main objective of the present work is to study the effects of hydrostatic pressure applied to the samples under varying excitation powers.

## 2. High-Pressure Study of the Electric Field in the Well

In [26], a novel high-pressure method for estimating the magnitude of the internal electric field (*F_int_*) in InGaN/GaN LEDs and LDs was proposed. The pressure coefficient of electroluminescence energy (*dE_EL_*/*dp*) was analyzed in InGaN/GaN LEDs as a function of driving current density. The application of hydrostatic pressure leads to an almost linear increase in the piezoelectric field in the quantum well (QW), as demonstrated in [24,25]. The pressure coefficient of the emission energy, i.e., *dE_EL_*/*dp* or *dE_PL_*/*dp*, analyzed for varying laser power density (LPD), provides insight into the evolution of *F_int_*. A well-established reference point is linked to the complete elimination of *F_int_* from the active region of polar nitride QWs, since in that case the pressure coefficient of *E_EL_* or *E_PL_* equals the pressure coefficient of the emission energy of bulk In_x_Ga_1−x_N alloy [25]. Unlike heterostructures, these bulk alloys exhibit no built-in electric field. The dependence of emission energy (*E_PL_*) on external hydrostatic pressure as a function of indium content is well documented, ranging from approximately 40 meV/GPa for GaN, decreasing almost linearly to 25–27 meV/GPa for x ≥ 0.25 [25]. Comparing *dE_PL_*/*dp* in the studied quantum wells with the pressure coefficient of In_x_Ga_(1−x)_N alloys (with the same indium content as the QW) provides critical information that is difficult to obtain by other means. The following analysis underpins this approach.

The transition energy in QWs, influenced by the built-in electric field, can be expressed as follows [13,27]:

(1) $$E_{L}=E_{G}+E_{conf}^{e}+E_{conf}^{h}-eL_{QW}\times F_{int}-E_{exc}$$

where *E_G_* represents the bandgap of the QW material, *E_L_* corresponds to *E_EL_* or *E_PL_*, *E^e^_conf_* and *E^h^_conf_* denote the confinement energies of electrons and holes (measured from the lower or higher edge of the well, respectively), *L_QW_* is the width of the well, *F_int_* is the electric field, and *E_exc_* is the exciton binding energy. Since *E_G_* is independent of the electric field, the derivative of *E_L_* with respect to pressure (the pressure coefficient) can be written as a sum of two terms [13,28]:

(2) $$\frac{{dE}_{L}}{dp}=\frac{{dE}_{G}}{dp}+\frac{{dE}_{L}}{{dF}_{int}}\times \frac{{dF}_{int}}{dp}$$

In this formulation, it is assumed that the pressure-induced changes in all terms of Equation (1) (except the first one) are caused by the pressure-induced variation of the *F_int_*.

The first term in Equation (2), *dE_G_*/*dp*, describes the pressure coefficient of the bandgap of alloys, which has been measured for quasi-bulk In_x_Ga_(1−x)_N, where *F_int_* is zero [25]. The second term involves the product of the change in transition energy with respect to the built-in electric field, *dE_L_*/*dF_int_*, which is negative (the transition energy decreases as the field increases), and the change in the electric field with pressure, *dF_int_*/*dp* (which is positive and nearly constant with pressure, as shown in [26]). Consequently, this product leads to a reduction in *dE_L_*/*dp* relative to *dE_G_*/*dp*, a decrease that becomes more pronounced for wider quantum wells and higher indium content in the QW. In our simplified approach, we assume that even under screening, the above equation holds, though the magnitude of *F_int_* is reduced (in reality, under screening, the field is non-uniform).

In cases of partial screening of the piezoelectric field within the QW, the value of *dE_L_*/*dF_int_* will be lower than that in an unscreened well, as the impact of the field on *E_L_* is less significant at low field strengths compared to high ones. Ultimately, when the field is fully screened, the measured pressure coefficient *dE_L_*/*dp* of the studied QW approaches the value of *dE_G_*/*dp*. Thus, deviations in *dE_L_*/*dp* from values reported for bulk InGaN can be used as an indicator of the internal electric field present in the QW [26].

It should be mentioned that *dE_G_*/*dp* in Equation (3) denotes the pressure coefficient of InGaN alloy lattice matched to GaN substrate. It is slightly lower than the pressure coefficient of *E_G_* in unstrained InGaN, since the compressibility of GaN is lower than that of InGaN, and for biaxially strained InGaN, we have elongation along the c-axis. However, we checked that the differences in *dE_G_*/*dp* do not exceed 0.5 meV/GPa, so they are within the experimental uncertainty of this parameter (±2.5 meV/GPa).

## 3. Materials and Methods

### 3.1. Samples

The studied structures were grown by plasma-assisted molecular beam epitaxy using a VG V90 MBE reactor (VG Semicon, East Grinstead, West Sussex, England, UK) equipped with two Veeco RF plasma sources. The structures were deposited on bulk GaN substrates with a 200 nm GaN:Si (Si: 1 × 10^18^ cm^−3^) buffer layer. A 0.17Ga0.83N single quantum well was grown with thicknesses of 2.6 nm, 5.2 nm, and 10.4 nm and was embedded between 20 nm-thick In_0.02_Ga_0.98_N barrier layers on both sides. The growth was conducted in metal-rich conditions at temperatures of 730 °C and 650 °C, for the buffer layer and InGaN layers, respectively. Quantum well and the barriers were not intentionally doped. More details on the growth methodology can be found in Ref. [28].

The indium composition was determined based on prior calibration growths performed under identical conditions. These calibration samples consisted of periodic InGaN structures, for which the indium content could be more reliably evaluated (e.g., by XRD). Based on these calibration procedures, the growth parameters were adjusted and subsequently used for the fabrication of the investigated QWs. Therefore, the reported indium composition of x = 0.17 should be understood as an estimated value derived from these calibrated growth conditions. The uncertainty of this estimation is on the order of Δx = ±0.01.

The choice of three QW widths enabled us to study the evolution of the radiative recombination mechanisms with changes in QW width. The overlaps of electron and hole ground states are large in 2.6 nm well (0.19–0.39, depending on concentration of screening carriers), small in 5.2 nm well, and negligible in 10.4 nm well (10^−13^–0.0006), as shown in [29].

### 3.2. Photoluminescence Measurements

The PL was excited with a Skylark (Edinburgh, Scotland, UK) 320NX DPSSL laser (wavelength of 320 nm, 3.87 eV). A maximum power of 50 mW on the sample surface was used on an area of 4.3 × 9.5 µm^2^; therefore, with an estimated maximum power density of around 114 kW/cm^2^. Laser power density was tuned by an attenuator from a minimal to maximal value of 2 W/cm^2^ and 10^5^ W/cm^2^, accordingly. The emission from the samples was collected in a backscattering geometry and dispersed using a SPEX500 (Irvine, CA, USA) spectrometer equipped with a 1200 nm diffraction grating. The signal was detected with a HORIBA Syncerity (Irvine, CA, USA) charge-coupled device (CCD) camera with 26 μm × 26 μm pixel size. A 10 μm slit was used, with an integration time of 0.01 s for high excitation laser densities and 1 s for low excitation densities (spectra were normalized). PL emission is strong at low temperatures, while electroluminescence disappears (in *pin* diodes) due to freezeout of Mg acceptors.

Since the excitation energy is above the bandgap of the QW barriers and of the substrate, we can expect to observe PL emission from the QW, In_0.02_Ga_0.98_N barriers and GaN.

First, we studied the PL dependence on the exciting laser power density (LPD). The measurement temperature was 20 K. The second type of PL studies involved the application of hydrostatic pressure (at a temperature of 80 K). Pressure (up to 4.2 GPa) was applied in the Diamond Anvil Cell [27] filled with argon. It was calibrated with the standard ruby gauge.

Application of hydrostatic pressure allows tuning of two parameters in the studied structures: electric field and bandgap. The bandgap of In_0.17_Ga_0.83_N QW shows an increase with rising pressure at a rate of about 31 meV/GPa.

## 4. Results

### 4.1. Excitation-Power Dependence of PL at 20 K

**Figure 1:**
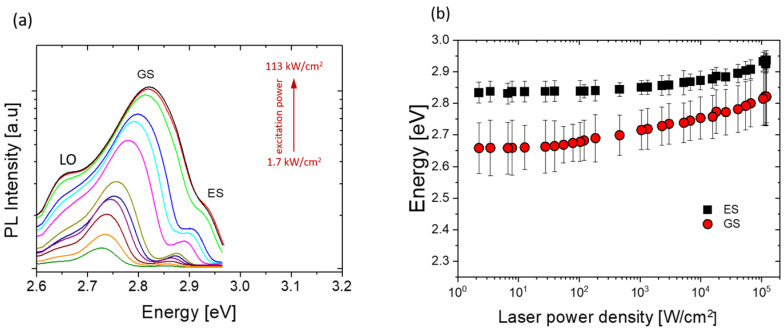
(**a**) PL spectra on a log scale for different excitation LPDs (starting from 1.7 kW/cm^2^ up to 113 kW/cm^2^) for sample A (2.6 nm well), (**b**) energy positions of three peaks versus LPD.

**Figure 2:**
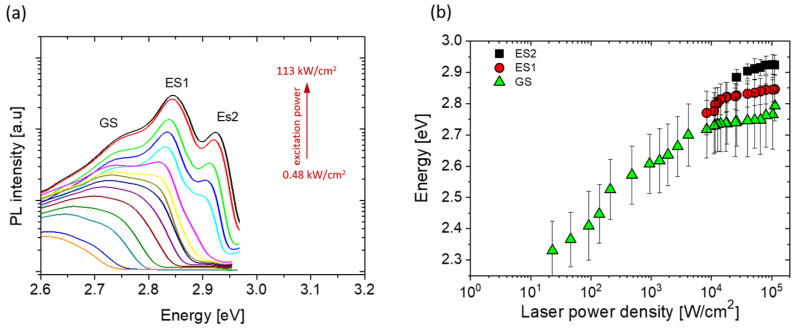
(**a**) PL spectra for different excitation LPDs (starting from 0.48 kW/cm^2^ up to 113 kW/cm^2^) for sample B (5.2 nm well), (**b**) energy positions of three peaks versus LPD.

**Figure 3:**
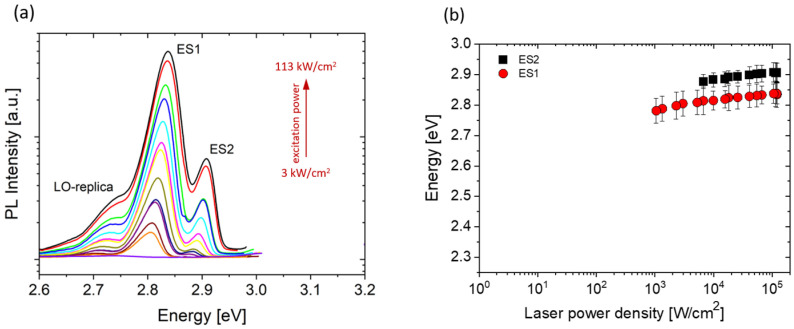
(**a**) PL spectra for different excitation LPDs (starting from 3 kW/cm^2^ up to 113 kW/cm^2^) for sample C (10.4 nm well), (**b**) energy positions of two peaks versus LPD.

Figure 1, Figure 2 and Figure 3 show the results of the PL spectra measurements on the studied QWs as a function of laser power density (LPD), at T = 20 K.

At the lowest LPD in Figure 1, one can observe two peaks at energies of about 2.73 and 2.87 eV. The lower-energy peak (2.73 eV) is associated with the ground state radiative transition: e1–h1. The higher-energy peak (2.87 eV) originates from a transition involving an excited state. The third peak at 2.65 eV becomes visible at higher LPDs. It is most probably the phonon replica of the main (e1–h1) transition.

With increasing LPD, the two observed peaks exhibit a blue shift (Figure 1b). This behavior is due to the screening of the built-in field by electron-hole pairs introduced by the exciting laser.

Figure 2a shows the dependence of PL spectra on the LPD in the single QW sample of 5.2 nm width (sample B). Three peaks appear consecutively in the spectra with increasing LPD. The lowest energy peak is associated with (e1–h1) radiative recombination involving a ground state transition. The energy of this peak exhibits a strong blue shift with increasing laser excitation (Figure 2b). At higher LPD, the energy shift saturates, which suggests a strong reduction in the built-in field.

Positions of the two higher-energy peaks exhibit much less sensitivity to the excitation power (Figure 2b). We assign the origin of these peaks to the contribution of the excited states. Both PL peaks can be observed at higher LPDs (due to more efficient screening), which leads to increased wave function overlap and greater occupation of the excited states.

Concerning the sample with a 10.4 nm QW width, we expect that electrons and holes generated by the laser beam tend to occupy opposite regions adjacent to the interfaces of this wide QW, so that for the ground state, the electron and hole wave function overlap is negligible. Increasing the number of carriers in the ground states generated by a laser beam (“dark charge”) starts to screen the field in the 10.4 nm QW, and above a certain concentration of the injected electron-hole pairs, the photoluminescence signal starts to appear. Please note that the emission appears above 1 kW/cm^2^.

Figure 3a illustrates PL spectra originating from three radiative transitions which cover the energy range between 2.6 and 3.0 eV. The lowest energy peak (with energies about 2.7–2.75 eV) is likely associated with the LO-phonon replica of the first excited state. Two peaks at about 2.8–2.9 eV are associated with the radiative transitions involving the excited states, ES1 and ES2 (Figure 3b). The energies of these two peaks are characterized by a very weak increase with laser power density. It strongly suggests almost complete screening of the QCSE in this sample.

It is worth pointing out that the behavior of photoluminescence (studied here) and electroluminescence (see, e.g., [13,29]) is similar in In_0.17_Ga_0.83_N/GaN QWs and LEDs with single QWs (in spite of the differences in the luminescence generation mechanism).

### 4.2. PL Under Hydrostatic Pressure at 80 K

High-pressure investigations of the three studied QWs with different widths were performed using the diamond anvil cell (DAC) technique at 80 K. The cell was filled with liquid argon, and the ruby fluorescence was used to calibrate the pressure.

**Figure 4:**
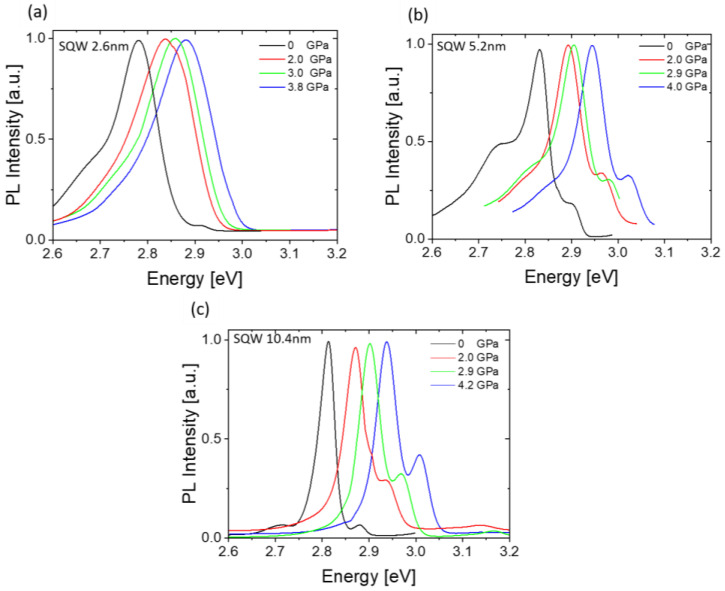
Pressure dependence of PL spectra for Sample A (**a**), Sample B (**b**) and Sample C (**c**) for the excitation power of 112 kW/cm^2^.

**Figure 5:**
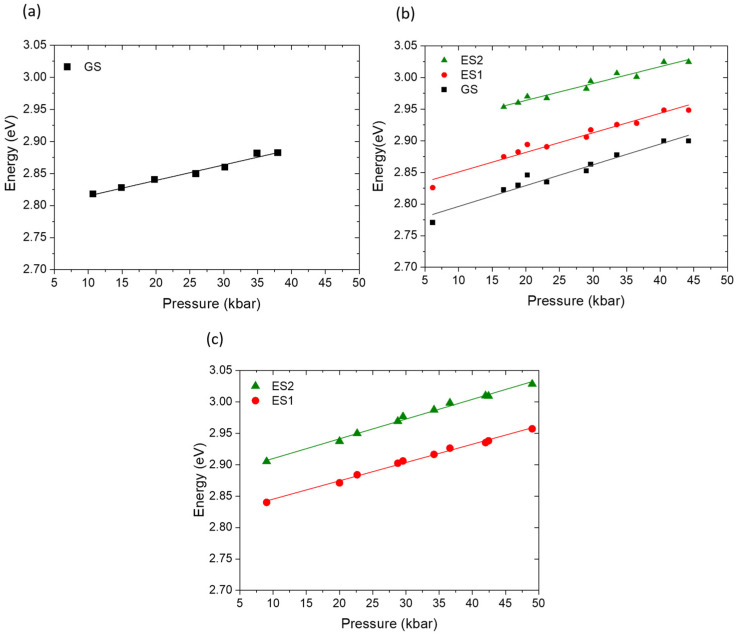
Pressure shift of the emission energies in Sample A (**a**), Sample B (**b**), and Sample C (**c**).

Three sets of normalized photoluminescence spectra at increasing pressures up to about 4 GPa (at a temperature of 80 K) are shown in Figure 4a–c. The spectra shift to higher energies with increasing pressure. This shift is approximately linear in the applied pressure range (4 GPa), as shown in Figure 5.

**Figure 6:**
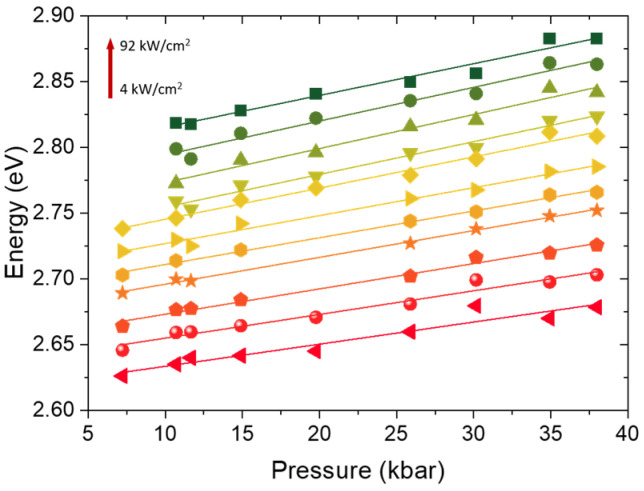
Pressure shift of the emission energy in Sample A for different excitation powers.

Similar measurements of the PL spectra as a function of pressure were performed for excitation power densities ranging from 2 to 10^5^ W/cm^2^ (Figure 6). The error bars in these figures are omitted for clarity, but they are the same as in Figure 1, Figure 2 and Figure 3.

**Figure 7:**
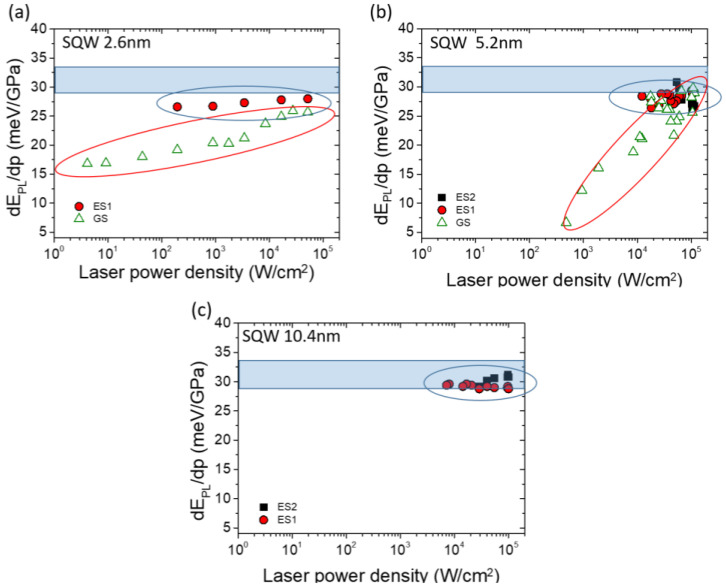
Pressure coefficients *dE_PL_*/*dp* versus LPD for Sample A (**a**), Sample B (**b**), and Sample C (**c**).

**Figure 8:**
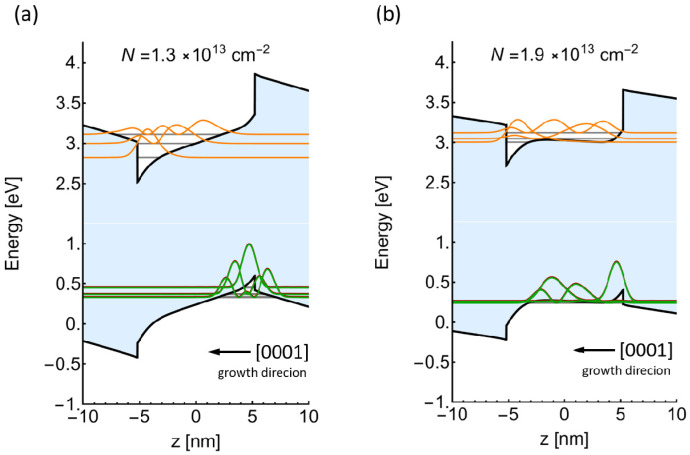
Wavefunctions for several bound states in the 10.4 nm QW (Sample C) at two carrier concentrations. (**a**) *N* = 1.3 × 10^13^ cm^−2^; (**b**) *N* = 1.9 × 10^13^ cm^−2^.

From these measurements, the pressure coefficient *dE_PL_*/*dp* has been obtained and is shown as a function of power density in Figure 7. The blue stripe, covering the range between 28.5 and 33.5 meV/GPa, corresponds to the bulk pressure coefficient of In_0.17_Ga_0.83_N. For the values of QW pressure coefficients in this region, we can infer that the electric field vanishes (in most of the well, see Figure 8). Below this range, the presence of the built-in field (increasing with pressure) reduces the pressure coefficient. The width of the blue stripe reflects the uncertainties in the determination of some parameters of the In_0.17_Ga_0.83_N/GaN QW samples (e.g., the correct indium content in the studied QW or the value of the pressure coefficient of bulk In_0.17_Ga_0.83_N).

Sample A, in the applied range of laser power density (2–10^5^ W/cm^2^), demonstrates the dominant contribution to photoluminescence originating from ground states (GS—green triangles). The excited state is barely visible at 80 K due to broadening of the lines. Increase in *dE_PL_*/*dp* with LPD corresponds mostly to the screening of the built-in field.

Figure 7b illustrates the evolution of the pressure coefficient of the three states observed in sample B (5.2 nm QW width). Pressure coefficient of the ground state (green triangles) increases with the applied LPD much more rapidly than in Sample A. This is because QCSE is stronger in a wider well, and the effect of screening is also stronger. The PL originating from the first and second excited states appears at two orders higher magnitude of LPD than for the ground state. It is due to the higher QCSE, i.e., lower electron-hole wave functions overlap in Sample B. The higher value of LPD increases the number of electron-hole pairs injected into the QW, creating the “dark-charge”, active in screening the built-in field without generating photoluminescence, for LPD up to 5 × 10^2^ W/cm^2^. At an LPD of about 10^4^ W/cm^2,^ two PL peaks related to the excited states start to appear. In this LPD range, the pressure coefficients of all three observed PL peaks are slightly lower than 31 meV/GPa. It corresponds to the presence of a very low built-in field.

Sample C (10.4 nm QW width) does not show PL emission related to the ground state transition e1–h1. Two excited states contribute to the PL signal at about 2.8 eV and 2.9 eV, and their observation requires LPDs higher than about 8 × 10^3^ W/cm^2^ (the first excited state) and 3 × 10^4^ W/cm^2^ (the second excited state). The resulting pressure coefficients in sample C (about 29–30 meV/GPa) show values comparable to those of bulk In_0.17_Ga_0.83_N. Thus, we can state that high-pressure behavior of these radiative transitions shows that Sample C illuminated with LPD higher than about 8 × 10^3^ W/cm^2^ (within the experimental error) is characterized by the absence of a built-in electric field in the central region of the well. This efficient screening of the electric field is due to the presence of a sufficient concentration of “dark charge” in the QW close to the interfaces (see Figure 8).

### 4.3. Simulation of the Pressure Dependence of PL Spectra

We simulate the radiative transitions in the three samples using the method described in [23,30]. For a given carrier concentration, the potential profile of the quantum well is determined by self-consistently solving the Poisson and Schrödinger equations, including the polarization charges and mobile charges. The valence band dispersion is described by the 6 × 6 kp Hamiltonian, and the conduction band by the spin-degenerate parabolic band. The band offset values were taken as *E_v_*/*E_c_* = 0.2/0.8. The differences in effective masses between the well and the barrier material are neglected. The model yields the dispersion of the heavy-hole band *E_hh_*(*k*), light-hole band *E_lh_*(*k*), crystal-field split-off band *E_cf_*(*k*) and the conduction band *E_c_*(*k*), where *k* is the in-plane wavevector. The wavefunctions for each band are determined in terms of the Bloch states at *k* = 0. The carrier concentration (equal for electrons and holes) determines the positions of the Fermi levels in the conduction and valence bands.

The example of wavefunctions for the 10.4 nm well (Sample C) for two concentrations of injected carriers is shown in Figure 8. At high carrier concentration, the electric field in this wide well is screened, causing increased wavefunction overlap for excited states. Meanwhile, the ground states for electrons and holes remain spatially separated.

Having determined the energy of bound states in the well, the overlaps between valence and conduction band states <e_i_|h_j_> are calculated for each pair of occupied (by electrons and by holes) states. Due to the strong electric field, all transitions (i,j) are allowed, with their strength dependent on <e_i_|h_j_> and on the occupation factors. In practice, it is sufficient to consider four electronic states and ten hole states. In most cases, no higher states are confined in the QW potential. The transitions are homogeneously broadened by the parameter *E_hom_*, which is estimated to be 25 meV at room temperature and about 10 meV at low temperatures (80 K and below). Even though the laser excitation occurs in TE mode (electric field in the plane of the well), we assume that carriers relax to the bottom of the band with random polarization and recombine, generating both TE and TM light, depending on the valence subband involved. At *k* = 0, TE-emission arises from heavy- and light-hole bands, and TM-emission from the crystal-field split-off bands. The spontaneous emission rate r(ħω) is calculated by summing up all transitions (vertical in *k*) between occupied conduction and valence states. The weak point of the simulation is the uncertainty of many important parameters of GaN and InGaN, like spontaneous polarization values, built-in electric fields, piezoelectric constants, etc.

On the simulated spectra (shown further in Section 4.4), each transition e_i_-h_j_ is shown by a color dot: the dot height shows the transition strength (dependent on the overlap and occupation of the states), while the color represents the initial state e_i_ (for example, the blue color denotes transitions from e_1_ to subsequent h_j_ states, j = 1, 2, 3…). For example, for the narrow well (Sample A), the spectrum is dominated by the transitions from the ground state e_1_; the transitions to hh_n_ and lh_n_ are so close that we denote them with the same symbol (lh1 + hh1 is called h1, lh2 + hh2 is called h2, etc.).

**Figure 9:**
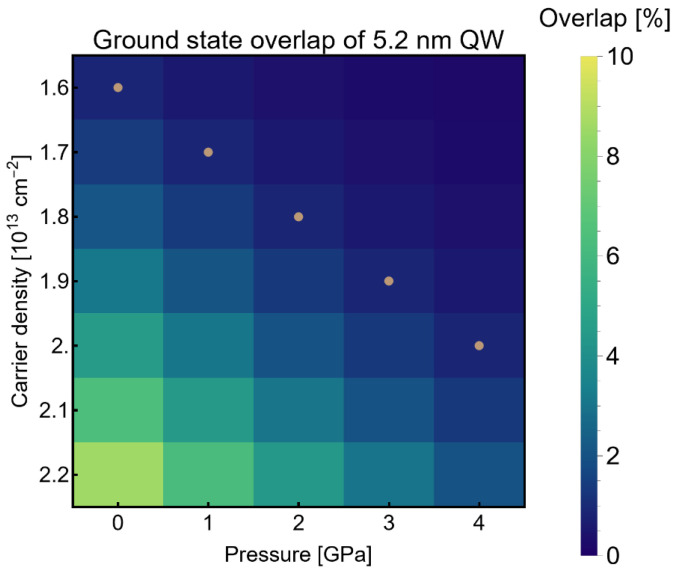
Squared wavefunction overlap for the ground states of electrons and holes in the 5.2 nm In_0.17_Ga_0.83_N/GaN well as a function of pressure and carrier density. The dots show the points of constant overlap |<e1|h1>|^2^ = 0.8%.

The electric field in In_x_Ga_1−x_N/GaN quantum well increases with hydrostatic pressure due to the increase in in-plane compressive strain [24]. We used the expression derived in [24] for the electric field *F_x_* as a function of composition *x* and pressure *p*:

(3) $$F_{x}\left(p\right)=\left(15+0.64p\right)x$$

where *p* is in GPa and the field is in MV/cm. Here we use the positive sign for pressure (it was negative in [24] to match the sign of deformation). In our case, *x* = 0.17, so the field at ambient pressure is about 2.5 MV/cm, and it increases approximately by 0.11 MV/cm per GPa. We also have to include the pressure increase in the bandgap in In_0.17_Ga_0.83_N, which is about 31 meV/GPa. We assume that all band structure parameters do not change with pressure (except for the bandgap). An open question is the pressure dependence of carrier concentration in the well under constant illumination. We assumed that the carrier density would increase under pressure since the field increases and the recombination rate (both radiative and non-radiative) should decrease. In order to obtain the approximate n(p) dependence, we calculated the overlap of ground state wavefunctions as a function of pressure and carrier density. Charge in the ground state is the main contributor to screening since electrons and holes are separated much more than in excited states. This is illustrated in Figure 9, where the squared overlap of the ground states is shown as a function of pressure and carrier density for the 5.2 nm well. The colors, ranging from yellow through green to blue, indicate a decreasing overlap (i.e., recombination) with increasing pressure and for decreasing charge density.

Since the ambient pressure PL spectrum was fitted with n = 1.6 × 10^13^ cm^−2^, we calculated the corresponding overlap and kept it constant under pressure. This is shown by dots in the figure. This means the carrier density should increase from 1.6 × 10^13^ cm^−2^ up to 2 × 10^13^ cm^−2^ at the pressure of 4 GPa. Similar analysis was performed for the 2.6 nm and 10.4 nm wells. The pressure variation in concentration is shown in Table 1.

### 4.4. Comparison with the Experiment

**Figure 10:**
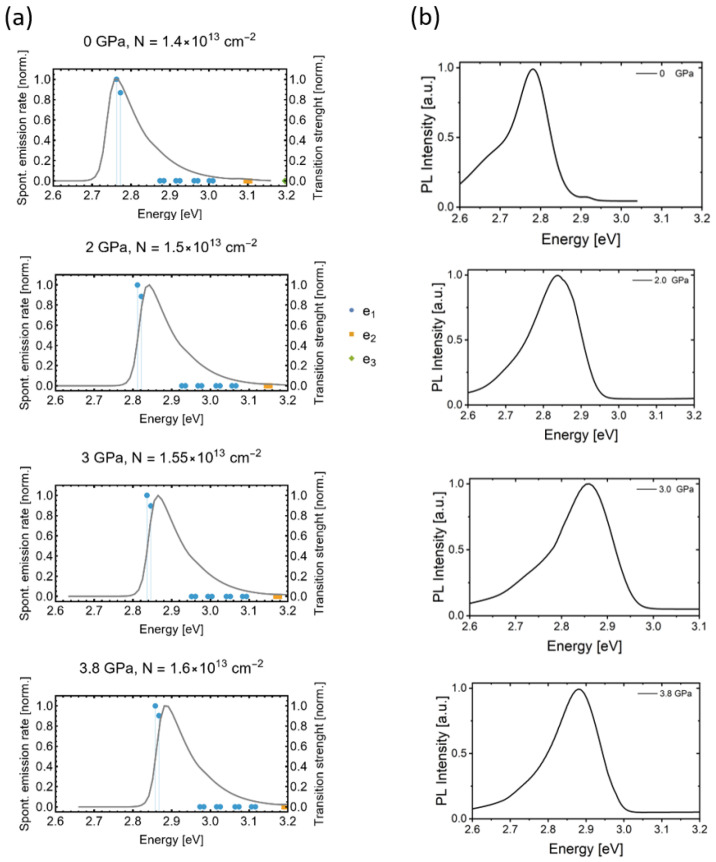
Theoretical spectra (**a**) compared to experimental spectra (**b**) for Sample A for selected pressures.

**Figure 11:**
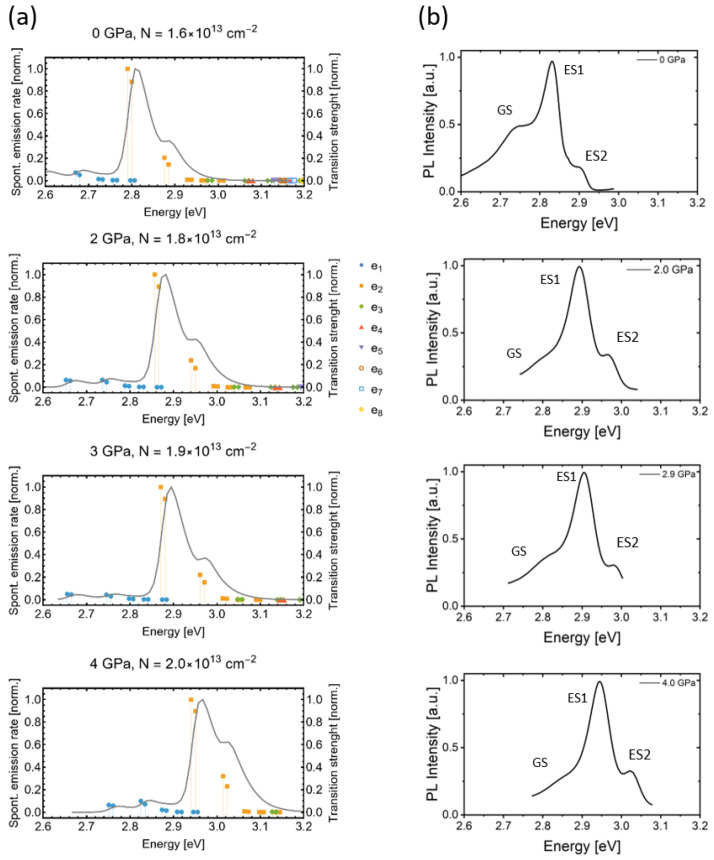
Theoretical spectra (**a**) compared to experimental spectra (**b**) for Sample B for selected pressures.

**Figure 12:**
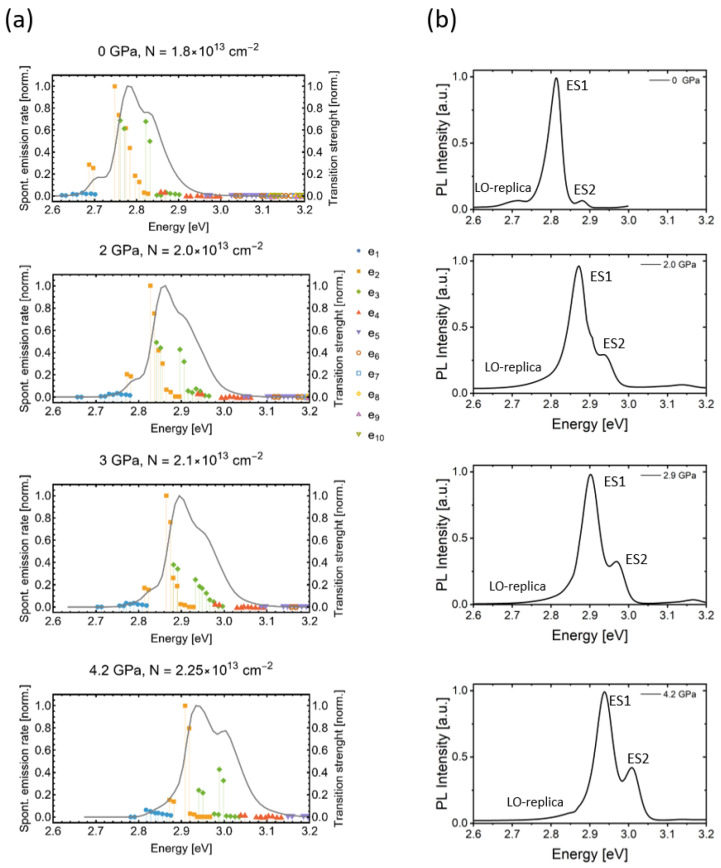
Theoretical spectra (**a**) compared to experimental spectra (**b**) for Sample C for selected pressures.

The concentrations in Table 1 have been used for the simulation of experimental spectra (shown for different pressures in Figure 10, Figure 11 and Figure 12 together with the corresponding experimental spectra).

In Sample A, the main peak is due to the e1–h1 transition. The higher energy transition (e1–h2) is only observed in the experiment at ambient pressure. The experimental spectra show a low-energy tail, which may be due to phonon replica.

In Sample B, the experimental spectra show a weak ground state transition and two excited-state transitions. The dominant peak is attributed to the e2–h1 transition, and the higher-energy peak to the e2–h2 transition.

In Sample C, we observe three peaks at low pressure (e2–h1, e2–h2 with a contribution of e3–h1, and e3–h2) and two peaks at high pressure (e2–h2 and e3–h2). The calculated spectra demonstrate that an experimental peak may be composed of several transitions. For the widest well, we also note that the ground state transition (blue dots) is absent due to negligible overlap, as shown in Figure 8b. This is consistent with the idea of dark charge, which only contributes to the screening of the electric field.

**Figure 13:**
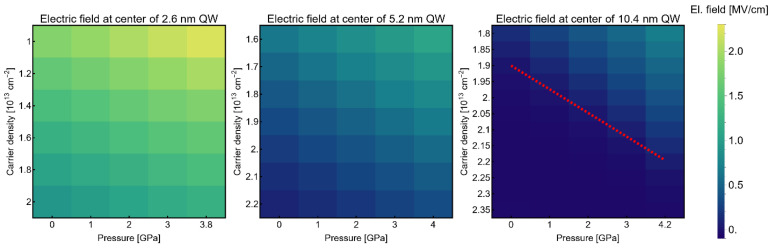
Color diagrams showing the electric field at the center of the well for three samples as a function of carrier density and pressure. The color scale is displayed in vertical bars. The red line is the border between the zero and the nonzero fields in the well.

Finally, it is interesting to show the effect of pressure and carrier concentration in our three samples using color diagrams, which display the value of the electric field in the well as a function of these two parameters. The electric field in the middle of the well increases with pressure and decreases with increasing concentration. The red dashed line for Sample C is the zero-field limiting line, corresponding to efficient screening of the built-in field (see Figure 13). For carrier concentrations and pressures below this line, the field is screened (in the middle of the well). Such “full” screening was only observed in the widest well (within the range of experimentally available LPDs). Crossing the red line represents the transition from a 2D-like system (separated eigenstates in the well) to a 3D-like system (where the excited states form a quasi-continuum). This has been analyzed in [23] as a function of carrier density; here, we show that it can be done using pressure.

## 5. Summary and Conclusions

We show different radiative recombination channels of single quantum wells with widths of 2.6, 5.2 and 10.4 nm. Evolution of PL energy depends strongly on the intensity of the PL-exciting laser. QW of 2.6 nm shows in the entire excitation range the PL signal originating from ground and first excited states (at 20 K). The energy of both states increases slowly with increasing LPD. Screening of the built-in polarization field (responsible for this change) is not very effective in this narrow well. Pressure coefficients of the emission are much lower than for bulk In_0.17_Ga_0.83_N. The excited state transition is barely visible at 80 K due to the broadening of the lines.

In a 5.2 nm QW, three electron-hole radiative recombination channels are involved. One observes, however, that the threshold LPDs for their appearance increase in the sequence: ground state, first excited and the second excited transition. It shows the contribution of carriers injected into the ground state, screening the built-in electric field more effectively than in the case of a narrow well. The pressure coefficient of the ground state transition is low at low LPDs but becomes close to that of bulk In_0.17_Ga_0.83_N at the highest LPDs. This implies almost complete screening of the electric field at such high LDPs.

In the QW of 10.4 nm width, the PL appears at the highest density of injected carriers, since it requires the formation of a sufficient density of “dark charge” screening the electric field. PL originates from two excited-state transitions. PL energy weakly increases with LPD. The pressure coefficients of emission lines are similar to those of bulk In_0.17_Ga_0.83_N. This demonstrates full screening of the field in most parts of the well (Figure 8). Therefore, high-pressure measurements (the dependence of pressure coefficients on LPD) provide additional evidence that the electric field in wide wells can be completely screened by free carriers in the ground state, allowing efficient recombination between excited states.

Theoretical analysis of the effect of pressure and concentration on the band structure has been performed and reasonable agreement with the experiment has been achieved. Simulations of emission spectra allowed us to identify electron and hole states contributing to the peaks observed in the experiment.

## Figures and Tables

**Table 1:** Carrier concentration (in 10^13^ cm^−2^) versus pressure in three samples.

Pressure [GPa]	0	1	2	3	3.8	4	4.2
Concentration in Sample A	1.4	1.453	1.505	1.558	1.6	1.611	1.621
Concentration in Sample B	1.6	1.7	1.8	1.9	1.98	2	2.02
Concentration in Sample C	1.8	1.91	2.01	2.121	2.207	2.229	2.25

## Data Availability

The raw data supporting the conclusions of this article will be made available by the authors on request.
